# Genetic background and window of exposure contribute to thyroid dysfunction promoted by low-dose exposure to 2,3,7,8-tetrachlorodibenzo-*p*-dioxin in mice

**DOI:** 10.1038/s41598-018-34427-2

**Published:** 2018-11-05

**Authors:** Carla Reale, Immacolata Porreca, Filomena Russo, Maria Marotta, Luca Roberto, Nicola Antonino Russo, Emanuele Carchia, Massimo Mallardo, Mario De Felice, Concetta Ambrosino

**Affiliations:** 1 IRGS, Biogem, Via Camporeale, 83031 Ariano Irpino, Avellino, Italy; 2 Molecular Medicine and Medical Biotechnologies, University of Naples “Federico II”, 80131 Naples, Italy; 3 IEOS-CNR, Via Pansini 6, 80131 Naples, Italy; 4 Department of Science and Technology, University of Sannio, via Port’Arsa 11, 82100 Benevento, Italy

**Keywords:** Thyroid Dysfunction, Longer Exposure Times, Fetal Exposure, Thyroid-disrupting Chemicals (THDC), TP53 Transcription, Environmental impact, Thyroid diseases, Risk factors

## Abstract

Genetic and environmental factors contribute to thyroid diseases. Although still debated, 2,3,7,8-tetrachlorodibenzo-*p*-dioxin (TCDD) is thought to induce thyroid dysfunction in humans and rodents. The data here reported point out the contribution of the exposure window and genetic background in mediating the low-dose TCDD effects on thyroid. Indeed, early (from E0.5 to PND30) and low-dose (0,001 μg/kg/day) TCDD exposure reduced the circulating fT4 and altered the expression of thyroid specific transcripts. The role of genetic components was estimated monitoring the same markers in *Pax8*^+/−^ and *Nkx2-1*^+/−^ mice, susceptible to thyroid dysfunction, exposed to 0, 1 μg/kg/day TCDD from E15.5 to PND60. Haploinsufficiency of either *Pax8* or *Nkx2-1* genes exacerbated the effects of the exposure impairing the thyroid enriched mRNAs in sex dependent manner. Such effect was mediated by mechanisms involving the Nkx2-1/p53/p65/IĸBα pathway *in vitro* and *in vivo*. Foetal exposure to TCDD impaired both thyroid function and genes expression while thyroid development and differentiation did not appear significantly affected. In mouse, stronger effects were related to earlier exposure or specific genetic background such as either *Pax8 or Nkx2-1* haploinsufficiency, both associated to hypothyroidism in humans. Furthermore, our data underline that long exposure time are needed to model *in vitro* and *in vivo* results.

## Introduction

Genetic and environmental factors concur to the development of several pathologies including thyroid diseases^1^. The incidence of these pathologies is growing worldwide^2^ and environmental factors, other than iodine deficiency, have been implicated. Genetic factors importantly influence both normal and pathological thyroid functions. Studies on congenital hypothyroidism (CH) have identified different genes (*Pax8, Nkx2-1*, etc.) as key players in thyroid organogenesis highlighting, moreover, few differences between mouse and human^3^. Several environmental pollutants, defined THyroid Disrupting Chemicals (THDC), are able to compromise the thyroid functionality acting at different levels on the hypothalamic–pituitary–thyroidal (HPT)-axis in humans and in animal models. Indeed, early-life exposure to THDC might impair thyroid tissue organization and/or the homeostatic regulation of thyroid adaptive processes in rodents^4,5^. Up to now the data obtained in animal model have been discontinuously confirmed in humans.

The 2,3,7,8-Tetrachlorodibenzo-p-dioxin (TCDD) is an environmental, ubiquitous and low-level contaminant, the most toxic among the polychlorinated dibenzodioxins (PCDDs), frequently retrieved at low dose in human fluids. TCDD is an endocrine disruptor exerting a pleiotropic activity by mechanism involving the Aryl-Hydrocarbon-Receptor (AhR) networks in human and animal models^6^. The AhR pathways are activated by chemicals similar to TCDD also in thyrocytes^7^. Although strongly debated, epidemiological^8–10^ and experimental^11^ studies suggest that TCDD and PCBs impair blood thyroxine (T4) and triiodothyronine (T3) levels. Exposure to TCDD has been shown to affect thyroid morphology and functionality in rodents^12,13^, with rats being more sensitive than mouse^14^.

The TCDD activity on the HPT axis observed in rodents has been only sporadically confirmed in humans^15^. This might be due to the variability of the genetic background of individuals, as opposed to the homogeneous genotype of the animal strains used in laboratory, and the relevance of the exposure window clearly evaluable only in animal models. Although pivotal in defining the risk associated to the environmental TCDD exposure, both aspects need further investigation. Indeed, researchers have poorly investigated the role of specific gene mutations, not having effect or inducing sub-clinical thyroid dysfunction, in exacerbating the TCDD activity. Furthermore, the thyroid-specific mechanisms of TCDD toxicity are not clarified yet. Their investigation is complicated *in vivo* because TCDD exerts its effects on HPT axis by the direct activity on the pituitary, on the thyroid gland or on both by the pituitary/thyroid feedback loop. Despite that, the effects of TCDD exposure on cellular models of thyrocytes have been assessed discontinuously.

We present data obtained both in mice and *in vitro* (in FRTL5, rat thyroid follicular cells) suggesting that low-dose TCDD carries out a direct effect on thyrocytes by a mechanism involving NF-κB pathway. We show that different TCDD doses, windows of exposure, genetic factors and, finally, sex are involved in mediating the impact of foetal TCDD exposure on the thyroid function.

## Results

### Exposure to low-dose TCDD since the conception induces phenotypic and molecular signs of hypothyroidism by a mechanism involving the Nkx2-1/p53/p65/IκBα pathway

Effects of early maternal exposure to TCDD on thyroid hormones (TH) status of dams and their offspring have not been detailed, as recently done for other chemicals^16,17^, because of the stressing administration routes usually adopted for TCDD administration (*i.e*. gavage). Intending to focus our attention on a “real exposure scenario”, we exposed C57BL/6 mice from E0.5 to the weaning by administration of food medicated with TCDD (TCDD-food) to pregnant dams and, then, feeding directly the offspring till the sacrifice (PND 30). A pilot experiment was done to verify the foetal toxicity of TCDD dosed at 0,1, 0,01 and 0,001 μg/kg/day. We did not obtain pups by pregnant dams exposed to 0, 1 μg/kg/day and 0, 01 μg/kg/day TCDD, in agreement with previous report showing that TCDD, dosed in similar range, exerted a toxic activity mainly on the pre-implantation embryos^18^.

Hence, TCDD 0,001 μg/kg/day exposure was adopted, a dose far below the ones used to investigate the TCDD-induced thyroid dysfunctions at later life stages (0,1 μg/kg/day)^16^. Treated dams had none statistical significant differences in the circulating free T4 (fT4, Fig. S1a) and in the expression of thyroid-stimulating hormone (*Tsh*) gene in pituitary (Fig. S1b), although TCDD activated the AhR pathway as suggested by the upregulation of its target *Cyp1A1* in thyroid (Fig. S1f) and pituitary (Fig. S1d).

**Figure 1:**
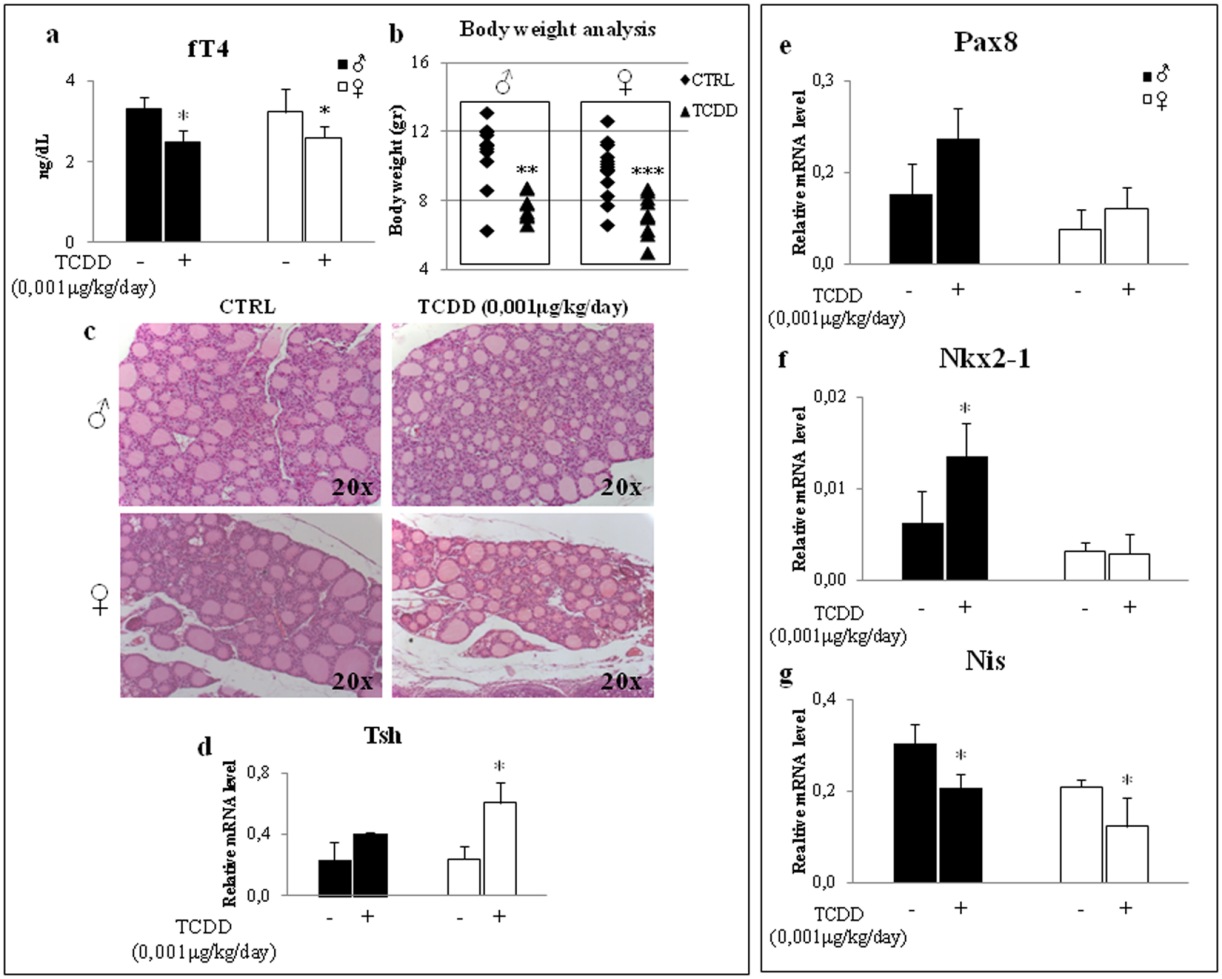
TCDD exposure since the conception impairs thyroid function. (**a**) fT4 serum levels were determined by ELISA in C57BL/6 males (black bar) and females (white bar) exposed at 0,001 μg/kg/day TCDD (TCDD) or not (CTRL), from E0.5 to PND30. (**b**) Body weight analysis of CTRL and TCDD-treated mice (male on the left and female on the right). (**c**) Hematoxylin and Eosin thyroid staining of CTRL- and TCDD-males (top panels) and females (bottom panels). (**d**–**g**) RT-qPCR analysis of *Tsh* transcript in pituitary and *Pax8*, *Nkx2-1* and *Nis* transcripts in thyroid of CTRL- and TCDD-males (black bar) and TCDD-females (white bar). Data are reported as means ± SD of *Gapdh* normalized-mRNA levels; n = 5 mice for each group and sex. *p-value < 0.05; **p-value < 0.01; ***p-value < 0.001 treated vs control mice.

The increase of *Cyp1A1* transcript was detected also in the thyroid (Fig. S2b) and pituitary (Fig. S2d) of the exposed offspring of both sexes in which TCDD exposure resulted in the reduction of circulating fT4 and body weight at the sacrifice (Fig. 1a,b, respectively). None major impact was evidenced in thyroid development as well as in thyroid morphology, as analysed by histochemistry (Fig. 1c). Although statistically significant only in females, *Tsh* transcript showed a trend towards the increase in the pituitary of TCDD treated mice (Fig. 1d). The expression levels of the sodium/iodide symporter (*Nis*), a TSH regulated gene^19^ also targeted by TCDD in primary thyrocytes^20^, and of its main transcriptional regulators *Pax8* and *Nkx2-1*, were analysed in thyroids of control and exposed mice. Despite the increase of *Tsh* mRNA (Fig. 1d) and of *Pax8* and *Nkx2-1* transcripts in males (Fig. 1e,f), the *Nis* mRNA decreased in both sexes (Fig. 1g). Being the level of thyroglobulin (*Tg*, Fig. S3a) and thyroperoxidase (*Tpo*, Fig. S3b*)* transcripts concordant with *Pax8* and *Nkx2-1* mRNAs, we suggested that TCDD might impair a pathway specifically involved in regulation of *Nis* expression. We supposed that NF-ĸB might be a mediator of TCDD activity in thyroid. Indeed, this transcriptional factor is involved in the regulation of thyroid specific genes expression^21,22^, mediates the TSH activity^23,24^ and interplays with AhR^25^. In addition, NF-ĸB is activated by pollutants able to increase the cellular reactive oxygen species (ROS), as TCDD^26^.

**Figure 2:**
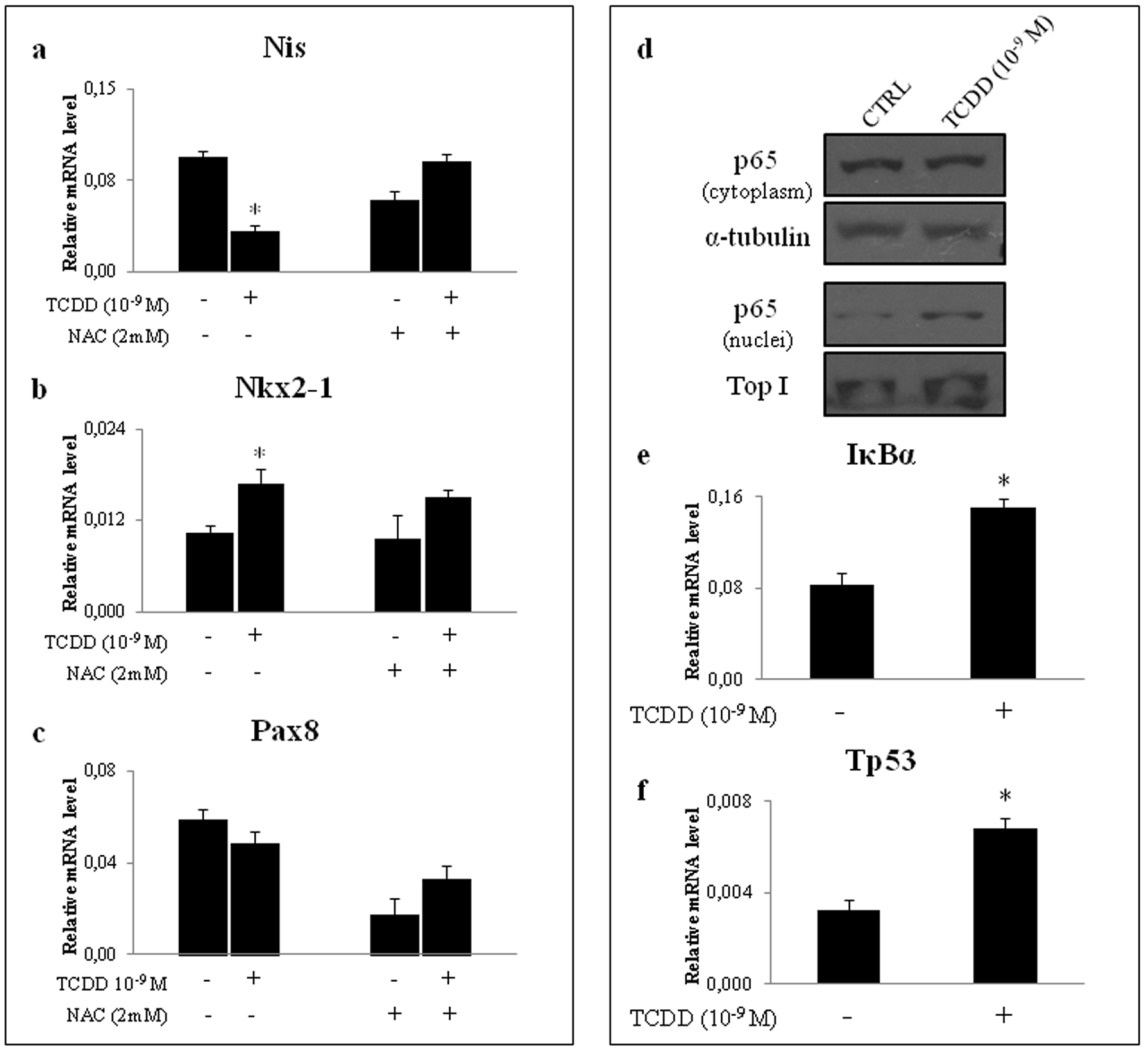
Oxidative stress contributes to alteration of thyroid-enriched transcripts in FRTL5 exposed to TCDD. (**a–c**) RT-qPCR analysis of *Nis* transcript (**a**) and thyroid specific regulators *Nkx2-1* (**b**) and *Pax8* (**c**) in FRTL-5 cells treated with TCDD 10^−9^ M for 24 hrs. When reported, cells were co-treated with NAC 2 mM for 24 hr. (**d**) Western blotting analysis of nuclear/cytosolic localization of p65 protein in FRTL-5 untreated (CTRL) and exposed to 10^−9^ M TCDD. β-Tubulin and Topoisomerase 1 were used as loading control of cytoplasmic and nuclear fraction, respectively. A cropped version of the image is shown (full-lenght blots are shown in Supplementary Fig. S7). Cytoplasmic and nuclear fractions were loaded on two different gels. Cellular level of *IκBα* and *Tp53* mRNAs (**e** and **f**, respectively) was assayed by RT-qPCR in CTRL- and TCDD-cells. Level of transcripts was determined after exposure to TCDD 10^−9^ M for 24 hr and reported as means ± SD of *Gapdh* normalized-mRNA levels of three independent experiments. *p-value < 0.05 compared with controls.

To test our hypothesis, we investigated the direct effects of TCDD on the immortalized rat follicular cell line FRTL-5, an evaluable model to study the response to low-dose environmental pollutants^27–29^. We exposed the cells to 10^−9^ M TCDD for 24 hrs, a setting previously reported to be associated with activation of AhR signalling and inhibition of *Nis* expression in primary thyrocytes^20^. The cellular content of *Nis* transcript was inhibited by TCDD as *in vivo* (Fig. 2a). The *Nkx2-1* expression was also increased whereas none significant effect was retrieved for *Pax8* messenger (Fig. 2b,c, respectively). TCDD induced the *Tg* and *Tpo* mRNAs as *in vivo* (see Supplementary Fig. S3c,d, respectively). To monitor the contribution of ROS production in the observed effect we co-treated the cells with N-acetyl-L-cysteine (NAC, 2 mM) to block ROS production. NAC treatment reversed the inhibition of *Nis* mRNA in TCDD-exposed cells, as well as the induction of *Tg* and *Tpo* transcripts (Fig. S3c,d), whereas none major effects was observed on the regulation of *Nkx2-1* expression (Fig. 2b). Finally, the level of *Pax8* transcript was reduced by NAC exposure and TCDD alleviated its inhibition (Fig. 2c).

The activation of NF-ĸB pathway was better characterized analysing the localization of its p65 subunit (p65) by western blotting. We found that the exposure to TCDD induced the p65 nuclear translocation (Fig. 2d). Concordantly, the expression of *IĸBα* mRNA, a NF-ĸB target gene, was increased (Fig. 2e). The reported activation of NF-ĸB together with the up-regulation of *Nkx2-1* mRNA suggested that TCDD might activate the Nkx2-1/p53/p65/IĸBα pathway, as previously described in lung carcinogenesis^30^. Therefore, we monitored the level of *Tp53* mRNA in the exposed cells retrieving its increase (Fig. 2f).

**Figure 3:**
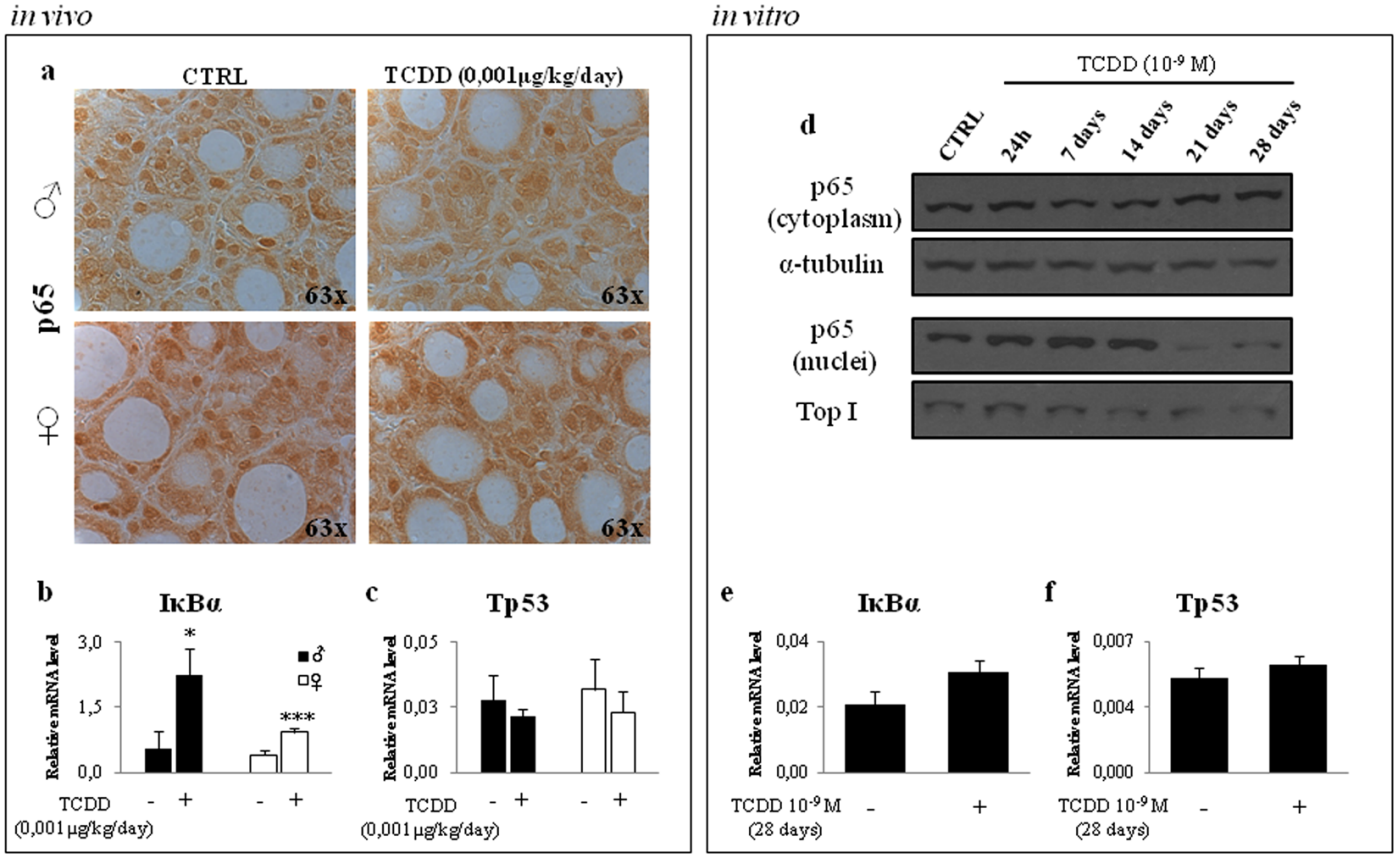
TCDD exposure induces the Nkx2-1/p53/p65/*IĸBα* pathway *in vivo* and *in vitro*. (**a**) Nuclear localization of p65 protein was assayed by immune-histochemistry in thyroids of control (CTRL) and 0,001 μg/kg/day TCDD treated-males (top panel) and TCDD-females (bottom panel) from E0.5 to PND30. Negative control was performed using purified IgG and it is shown in Fig. S3g. (**b**,**c**) Levels of *IκBα* (**b**) and T*p53* (**c**) transcripts were assayed by RT-qPCR in thyroid of CTRL- and TCDD-mice of both sexes (black bar for male, white bar for female). (**d**) Nuclear/cytosolic localization of p65 protein in FRTL-5 untreated (CTRL) and exposed to 10^−9^ M TCDD for 1-, 7-, 14-, 21-, 28- days was assayed by western blotting analysis. β-tubulin and Topoisomerase 1 were used as loading control of cytoplasmic and nuclear fraction, respectively. A cropped version of the image is shown (full-lenght blots are shown in Supplementary Fig. S7). Cytoplasmic and nuclear fractions were loaded on two different gels. (**e**,**f**) RT-qPCR analysis of *IĸBα* (**e**) and T*p53* (**f**) transcript levels in FRTL-5 cells treated with TCDD 10^−9^ M for 28days. *In vitro* data are reported as means ± SD of *Gapdh* normalized-mRNA levels of three independent experiments. *In vivo* data are reported as means ± SD of *Gapdh* normalized-mRNA levels; 5 mice for each group and sex were analyzed. *p-value < 0.05 and ***p-value < 0.001.

Considering that *Nkx2-1* mRNA showed a trend towards the induction in treated males, we verified if the Nkx2-1/p53/p65/IĸBα pathway might be active also in mice exposed to 0, 001 μg/kg/day from E0.5 to PND30 (TCDD-mice). Concordantly, with the induction of *Nkx2-1* transcript (Fig. 1f), *IκBα* mRNA increased in thyroids of TCDD-mice, although strongly in males (Fig. 3b). The p65 localization in the thyroids of control and TCDD-mice was assessed by immunohystochemistry. We observed the reduction of the p65 nuclear staining in thyroid of exposed animals of both sexes (Fig. 3a), which might be explained by the increased transcription of *IκBα* promoting its cytoplasmic retention. None difference was detected in *Tp53* transcripts (Fig. 3c). Since the *in vivo* data partially confirmed the *in vitro* results, we verified if this discrepancy might be related to a time-dependent activation of NF-ĸB signalling being longer exposure time applied *in vivo*. Therefore, we exposed the FRTL-5 cells to 10^−9^ M TCDD for 1-, 7-, 14-, 21- 28 days and nuclear/cytoplasmic localization of p65 protein was analysed by western blotting (Fig. 3d). The data evidenced that the increase of nuclear level of p65 was on-going till the day 14 and, then, it was reduced at the later time points. We measured the level of *Nis* mRNA and the transcripts of the Nkx2-1/p53/p65/IκBα pathway at this last time point. The level of *Nis* and *Nkx2-1* mRNAs was slightly affected in FRTL5 exposed to TCDD for 28 days compared to cell treated for 1 day (see Supplementary Fig. S4 and Fig. 2, respectively). The long exposure time (28 days) resulted in milder impairment of *IκBα* mRNA (Fig. 3e) and none major change was detected for *Tp53* transcript (Fig. 3f).

Overall the *in vivo* and *in vitro* data suggest that the early foetal exposure to low-dose TCDD affects thyroid function altering the expression of thyroid specific transcripts, mainly *Nis*, by a mechanism, at least partly, involving the Nkx2-1/p53/p65/IĸBα pathway differently in the early or late phase of the exposure.

### Haploinsufficiency of Pax8 and Nkx2-1 predisposes to impairment of thyroid function in TCDD exposed mice

**Figure 4:**
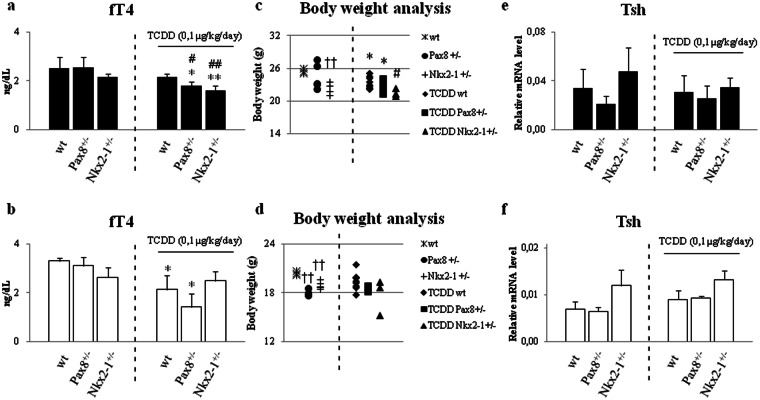
Characterization of thyroid function in mice with different genetic background or foetally exposed to TCDD. (**a**,**b**) ELISA assay of fT4 serum level was performed in C57BL/6 wild type (wt), *Pax8*^+/−^ and *Nkx2-1*^+/−^ mice (at left of the dashed lines) and in *wt*, *Pax8*^+/−^ and *Nkx2-1*^+/−^ treated with TCDD 0,1 μg/kg/day (at right of the dashed lines), males (**a**) and females (**b**). (**c**,**d**) Body weight analysis of *wt*, *Pax8*^+/−^ and *Nkx2-1*^+/−^ and TCDD-*wt*, -*Pax8*^+/−^ and -*Nkx2-1*^+/−^ mice, males (**c**) and females (**d**). (**e**,**f**) RT-qPCR analysis of *Tsh* in pituitary of *wt*, *Pax8*^+/−^ and *Nkx2-1*^+/−^ and TCDD-*wt*, -*Pax8*^+/−^ and -*Nkx2-1*^+/−^ mice, males (**e**) and females (**f**). Data are reported as means ± SD of *Gapdh* normalized-mRNA levels; n = 4 mice for each group. *p-value < 0.05; **p-value < 0.01 for statistical analysis of TCDD-exposed *vs* untreated mice having the same genotype; ^#^p-value < 0.05; ^##^p-value < 0.01 TCDD-exposed *vs* TCDD-wt mice; ^††^p-value < 0.01 unexposed mice of different genotype *vs wt* mice.

Thyroid diseases have a strong genetic component. In order to evaluate the role of the genotype in determining the effects of TCDD exposure, we used mice heterozygous for deletion of either *Pax8* (*Pax8*^+/−^) or *Nkx2-1* (*Nkx2-1*^+/−^). Haploinsufficiency of each of these genes associates with hypothyroidism in human and has slighter effects in mouse^31^. They might represent a good model to investigate the role of the genotype in the impairment of thyroid function by TCDD exposure. Indeed, the *Nkx2-1*^+/−^ mice had a slight reduction of the circulating level of fT4 (Fig. 4a,b, at left of the dashed lines), a trend toward the reduction in the body weight (Fig. 4c,d, left side of the graphs) and increased levels of *Tsh* mRNAs (Fig. 4e,f, at left of the dashed lines). None phenotypic difference was retrieved in the *Pax8*^+/−^ mice that showed a decreased level of *Tsh* transcript (Fig. 4e,f, at left of the dashed lines).

To assess the role of either *Pax8* or *Nkx2-1* haploinsufficiency in exacerbating the TCDD-induced thyroid dysfunction, we delayed the exposure time at E15.5, when the thyroid organogenesis is completed^19^. TCDD was dosed at 0, 1 μg/kg/day as previously done for this exposure window^16,32,33^. The impairment of thyroid function by TCDD in the new exposure condition was before tested in wild type C57BL/6 (*wt*). Pregnant females were fed with food medicated with TCDD (0, 1 μg/kg/day) since E15.5. The offspring was exposed by the mothers till the weaning and, then, by direct feeding till the sacrifice at PND60. TCDD exposure did not impair the levels of circulating fT4 or of the *Tsh* transcript of the mothers (Fig. S1g,h) although it resulted in the AhR activation, as documented by the increase of the *Cyp1A1* mRNA in their pituitary and thyroids and (Fig. S1l,n). *Cyp1A1* transcript was increased also in the thyroid (Fig. S5b,d) and pituitary (Fig. S5f,h) of the offspring. Circulating fT4 was weakly reduced in wt mice exposed to TCDD, although the difference was statistically significant only in females (Fig. 4a,b, at right of dashed line). Slight but not statistically significant differences could be detected in body weight and *Tsh* mRNA expression in the pituitary in wt mice (Fig. 4c–f, at right of dashed line). The reported results suggested that the figured exposure plan might highlight the role of genetic factors, predisposing to thyroid dysfunction, in worsening TCDD effects (*i.e*. the haploinsufficiency of either *Pax8* or *Nkx2-1)*. Two months old *wt* females were mated with males carrying the heterozygotic loss of function mutation of *Pax8* and *Nkx2-1* gene. Pregnant dams and offspring were exposed as reported above. Pups were genotyped at PND15 in order to conduct the phenotypic and molecular analysis of the TCDD activity considering genotypes and sex as important factors influencing thyroid dysfunctions.

**Figure 5:**
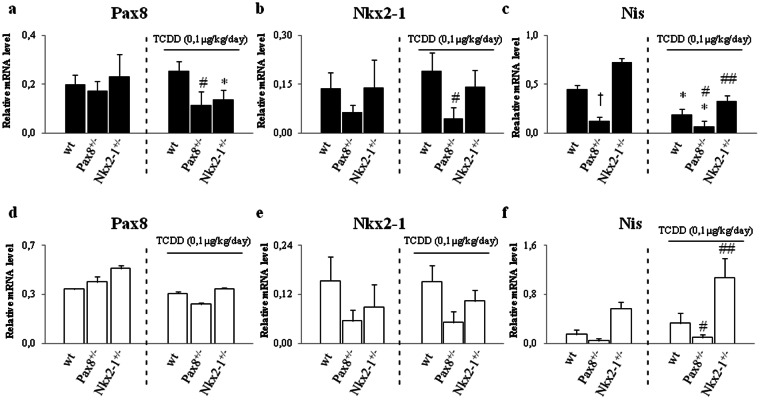
Haploinsufficiency of *Pax8* or *Nkx2-1* exacerbates the effects of TCDD treatment. Expression of thyroid specific transcripts in unexposed or 0,1 μg/kg/day TCDD exposed mice (from E15.5 to PND60) of each genotype. Thyroid *Pax8*, *Nkx2-1* and *Nis* transcripts were assayed by RT-qPCR in C57BL/6 wild type (*wt*), *Pax8*^+/−^ and *Nkx2-1*^+/−^ (at left of the dashed lines) and in TCDD-*wt*, -*Pax8*^+/−^ and -*Nkx2-1*^+/−^ (at right of the dashed lines), males (**a**,**b** and **c**) and females (**d**,**e** and **f**). Data are reported as means ± SD of *Gapdh* normalized-mRNA levels; n = 4 mice for each group. *p-value < 0.05; **p-value < 0.01 for statistical analysis of TCDD-exposed *vs* untreated mice having the same genotype; ^#^p-value < 0.05; ^##^p-value < 0.01 TCDD-exposed *vs* TCDD-wt mice; ^†^p-value < 0.05 unexposed mice of different genotype *vs wt* mice.

Circulating fT4 was reduced in statistically significant manner in both TCDD-*Pax8*^+/−^ and TCDD*-Nkx2-1*^+/−^ males versus (*vs*) TCDD-*wt* mice (Fig. 4a). Although TCDD exposure had an effect on the body weight, it was influenced by the genotype only in TCDD-*Nkx2-1*^+/−^ males *vs* TCDD*-wt* mice (Fig. 4c). Surprisingly, we observed less pronounced TCDD effects in females. TCDD exposure resulted in the reduction of circulating fT4 in TCDD-*Pax8*^+/−^ females *vs* TCDD*-wt* (Fig. 4b) but it did not affect the body weight at the sacrifice (Fig. 4d). None clear effect could be detected in pituitary level of *Tsh* mRNA (Fig. 4e,f), even in males in which the reduction of circulating fT4 was statistically significant. In order to molecularly characterize the effects of TCDD exposure, we analyzed the expression of thyroid specific transcripts and their upstream regulators in exposed animal of each genotype and sex. Focusing on the effect of the exposure, the data revealed that *Pax8* transcript was reduced in TCDD*-Pax8*^+/−^ males *vs* TCDD-*wt* mice (Fig. 5a) whereas *Nkx2-1* showed a trend towards the down-regulation in the TCDD-males of both genotypes, reaching the statistical significance only in the TCDD-*Pax8*^+/−^ males *vs* TCDD*-wt* mice (Fig. 5b). Concordantly, the inhibition of *Tg* was statistically significant only in TCDD-*Pax8*^+/−^ mice of both sexes *vs* TCDD-*wt* whereas *Tpo* was inhibited in TCDD-*Pax8*^+/−^ males (see Supplementary Fig. S3e,f). *Nis* mRNA was strongly inhibited in the TCDD-*Pax8*^+/−^ mice *vs* TCDD-*wt* whereas it was induced in TCDD-*Nkx2-1*^+/−^, both in males and females (Fig. 5c,f).

Overall, the data highlighted the interplay between TCDD, genetic factor and sexes and defined them as factors influencing the response to the TCDD exposure.

**Figure 6:**
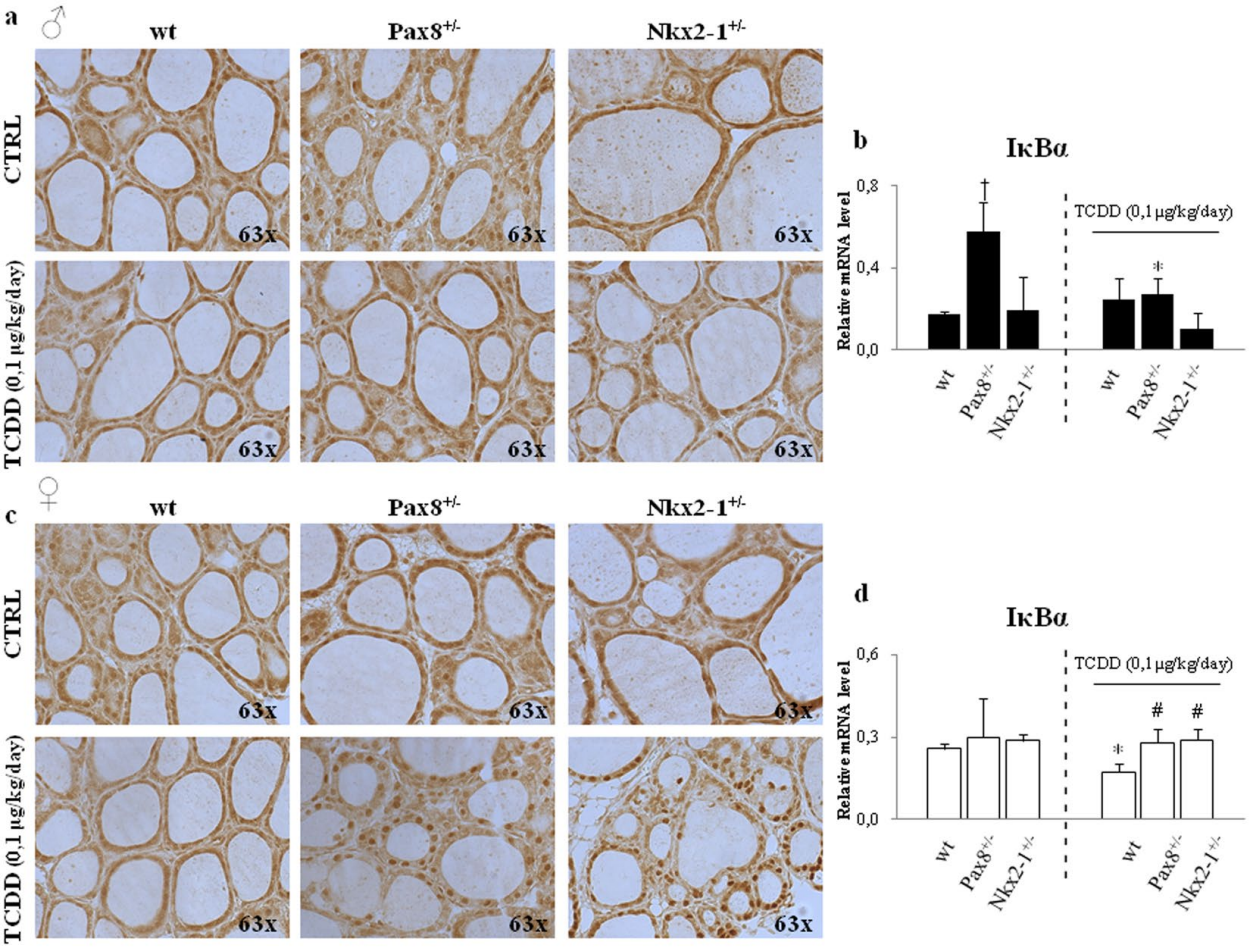
Activation of NF-κB pathway in *Pax8*^+/−^ or *Nkx2-1*^+/−^ exposed mice. (**a**,**c**) Immune-peroxidase analysis of p65 protein in thyroids of CTRL *wt*, *Pax8*^+/−^ and *Nkx2-1*^+/−^ and of TCDD-*wt*, -*Pax8*^+/−^ and -*Nkx2-1*^+/−^, males (**a**) and females (**c**) (n = 4 mice for each group and sex). Negative control was performed using purified IgG and it is shown in Fig. S3h. (**b**,**d**) RT-qPCR of *IκBα* gene in thyroid of C57BL/6 wild type (*wt*), *Pax8*^+/−^ and *Nkx2-1*^+/−^ (at left of the dashed lines) and in TCDD-*wt*, -*Pax8*^+/−^ and -*Nkx2-1*^+/−^ (at right of the dashed lines), males (**b**) and females (**d**). Data are reported as means ± SD of *Gapdh* normalized-mRNA levels; n = 4 mice for each group and sex. *p-value < 0.05 for statistical analysis of TCDD-exposed *vs* untreated mice having the same genotype; ^#^p-value < 0.05 TCDD-exposed *vs* TCDD-wt mice; ^†^p-value < 0.05 unexposed mice of different genotype *vs wt* mice.

Then, we analyzed the p65 nuclear localization by immune-histochemistry (Fig. 6a,c) and immunofluorescence (see Supplementary Fig. S6a,b) and the expression of its target *IκBα* (Fig. 6b,d). This transcript was regulated in sex dependent manner by TCDD exposure, being inhibited by TCDD exposure in wt exposed females (Fig. 6d) and showing a trend towards the up-regulation in exposed wt males (Fig. 6b). *IκBα* was not regulated by TCDD in *Pax8*^+/−^ and *Nkx2-1*^+/−^. The data were corroborated by the analyses of p65 localization by immune-hystochemistry showing that in TCCD-*Pax8*^+/−^ and TCDD-*Nkx2-1*^+/−^ females it was localized in the nucleus similarly to unexposed females of the same genotype and more than in TCDD-wt females. These lasts presented a reduced nuclear staining for p65 vs the unexposed wt-females. These last data might explain the milder effect of TCDD exposure in females. As expected, no changes were detected in *Tp53* mRNA levels (data not shown).

Overall, the above reported results underlined that foetal exposure to TCDD resulted in thyroid function impairment whose strength was genotype- and sex-dependent. Indeed, the TCDD exposure was worsened in genetic context characterized by haploinsufficiency of *Pax8*^+/−^ or *Nkx2-1*^+/−^, genetic conditions, associated with hypothyroidism in humans.

## Discussion

Although neonatal hypothyroidism has been associated with maternal exposure to TCDD in rodents, this suggestion is strongly debated in humans. The question has been reintroduced by a recent paper describing that the maternal exposure to TCDD resulted in a long-lasting, neonatal thyroid dysfunction in the highly exposed population of Seveso (Italy)^15,34^. In the 1976, approximately 30 kg of TCDD were released in area surrounding a trichlorophenol manufacturing plant in Seveso (Italy), determining the highest known exposure to TCDD in a residential population. The accident was monitored since the beginning to investigate the effects of TCDD exposure on hormonal axis at different ages. The women were followed through the Seveso Women’s Health Study, revealing that the serum TCDD levels were inversely correlated with total T4 levels in women exposed before the menarche in this population^15^. These results underscored that the impact on thyroid function of TCDD needs to be investigated with the aim to translate better the results from rodents to humans. This would imply both the consideration of realistic scenario of exposure, characterized by low-dose and foetal exposure of genetically different individuals, and the identification of thyroid molecular targets of TCDD activity that can be verified cross-species^35^. Here, we tried to address both aspects.

The possible impact of foetal TCDD exposure on thyroid function has been suggested since long time because the cleft palate^36^, a malformation associated to congenital hypothyroidism^37^, was recognized as a common feature of high-dose TCDD foetal exposure in C57BL/6 mice. Despite that, the TH status in exposed animals has not been specifically investigated, even in recent studies reporting the molecular mechanisms of TCDD activity^38^. Although very early foetal exposure (E0.5) to low-dose TCDD did not damage thyroid development, here we report its effects on the thyroid function evidenced by the reduction of circulating fT4 and body weight as well as the increased transcription of *Tsh* in pituitary in all the exposed mice. The observed effects on the pituitary are in agreement with previous report describing the TCDD impact on pituitary hormone transcripts, even if the Authors analyzed the effects on the hypothalamic–pituitary–gonadal axis (HPG axis) in different exposure settings^33^. Here we further suggest that the TCDD-induced thyroid dysfunction is mechanistically associated to effects, sometimes slight, on the transcription of several thyroid specific genes. As we have previously proposed for other EDCs, they might cumulatively promote the phenotypic effects *in vivo*^27,34^. Some molecular results underscore the peculiarity of TCDD exposure: *i.e*. the unexpected divergence between the *Tsh* transcript increase and *Nis* mRNA inhibition not involving *Tg* and *Tpo* mRNAs, whose transcription is also dependent by TSH. This suggests that TCDD alters a mechanism mediating specifically the regulation of *Nis* expression by TSH.

Considering that the pituitary/thyroid feedback loop complicates mechanistic analysis *in vivo*, we performed part of our study on FRTL-5, a cellular model different from the ones already adopted^20,39^. The reported data agreed with previous results that showed the inhibition of *Nis* transcript in primary thyrocytes exposed to TCDD, associated to activation of the AhR signalling. Using bioinformatics analysis, we failed in finding AhR binding sites in rat or mouse *Nis* promoter (data not shown). This suggested us that other transcriptional regulators enriched (*Pax8*, *Nkx2-1*, etc) or not (NF-κB, etc) in thyroid could have a role in mediating the TCDD thyroid effects. The thyroid gene expression is controlled by their specific combination, established by the respective promoters/enhancers organization^21,40^. Their exact combination is required to induce/inhibit gene expression. Here, we propose the role of p65 and Pax8 in the regulation of *Nis* expression by TCDD not excluding the AhR signalling, indeed activated in all the different exposure conditions as stated by up-regulation of *Cyp1a1* transcript. Activated AhR might contribute to p65 nuclear activation^25^ following TCDD exposure. Indeed, both proteins negatively regulate *Nis* expression in oxidative conditions, such as the iodide-induced ROS production, the last induced by TCDD in FRLT-5. The here retrieved p65 activation and I*κ*Bα overexpression has been positively associated to the increase of *Nkx2-1* transcript in lung^41^. These proteins with p53 participate to the Nkx2-1/p53/p65/I*κ*Bα pathway involved in lung carcinogenesis^30^. Here, we show that the same pathway is activated in TCDD-exposed FRTL-5 and in mice, although partially, suggesting a possible mechanism of TCDD-induced thyroid carcinogenesis in human^42^ and rodents^43^. Furthermore, the reported results underline that longer exposure times are required to model *in vitro* the *in vivo* response to EDCs, as we have previously shown^34^.

The *in vivo* data point to the sex as a factor influencing the response to TCDD. This is not a novelty as a sex dependent modification of liver transcriptome by TCDD has been reported^44^. Here, we show sex related differences in the TCDD regulation of thyroid-enriched mRNAs as well as of the involved molecular mechanisms. Indeed, *Nis* transcript could not be induced in TCDD-males because of the p65 inhibition whereas in TCDD-females because *Pax8* and *Nkx2-1* transcripts were not induced. Overall the *in vivo* and *in vitro* data suggest a direct, sex and time dependent impairment of Nkx2-1/p53/p65/IκBα axis by TCDD in thyrocytes. As said, its activation was partially confirmed *in vivo* suggesting that the longer exposure time applied in mouse might promote the compensation processes needed for surviving (*i.e*. inhibition of *Tp53* transcription, to allow the cell proliferation, or inflammation block). Hence, longer exposure times or higher TCDD dose could be needed for a stable activation of Nkx2-1/p53/p65/IκBα pathway *in vivo*. Both conditions characterize the accidental human exposure already associated to the increased incidence of thyroid cancer^45^. Its activation during the foetal life might promote the low-dose induced TCDD thyroid carcinogenesis^46^.

Evidences of long-term and low-dose effects of TCDD on human thyroid are debated. The contrasting results might be largely attributed to the genetic variability in human population and to the impossibility to control precisely the window, the route and the dose of exposure. It was already assumed that genetic factors influenced the response to TCDD because it was differently developed up to the strains adopted, being the C57BL/6 TCDD-sensitive and DBA resistant^47^. Heterozygous mutations of thyroid enriched master regulators, such as *Pax8* and *Nkx2-1*, have been associated to different grades of human hypothyroidism. Environmental factors could concur to exacerbation of their effects. The presented data corroborate this suggestion showing that TCDD exposure induces a thyroid dysfunction stronger in *Pax8*^+/−^ or *Nkx2-1*^+/−^ males *vs* wild type mice at phenotypic and molecular level. Indeed, haploinsufficiency of *Pax8*^+/−^ or *Nkx2-1*^+/−^ resulted in the inability to restore the normal thyroid specific regulation, as shown for *Tg* or *Nis* transcripts. Furthermore, the genotype affected in sex dependent manner the impairment of the Nkx2-1/p53/p65/IκBα pathway promoted by TCDD exposure, corroborating the hypothesis that it is a player of TCDD action in mouse thyroid.

## Conclusion

The reported *in vivo* data point to genetic factors in combination with the window of exposure and the sex as key players in the thyroid dysfunction following foetal exposure to low-dose TCDD. *In vitro* and *in vivo* evidences suggest the Nkx2-1/p53/p65/IκBα pathway, or its components, as mediator of TCDD effects on thyrocytes, here highlighted by the analysis of several molecular markers of thyroid activity. Thus, this report represents one of the few attempts to identify mechanisms of TCDD thyroid toxicity, showing the role of the *in vitro* studies in their identification.

Overall, the data here presented underline the complexity to dissect the effects of TCDD giving suggestion of the multiple factors, above all the single genotype, which should be considered in analysing them in humans and in animal models.

## Methods

### Cell culture and treatment

FRTL-5 cells were grown in Coon’s modified F12 medium (EuroClone) supplemented with 5% newborn bovine serum (HyClone Laboratories) and six hormones mixture^48^. Cells were exposed to TCDD (Chemical Research), dissolved in DMSO at a concentration of 100 μM then diluted at 1 × 10^−9^ M in culture medium. Controls were treated with vehicle alone. The ROS inhibitor, N-acetyl-L-cysteine (NAC, Sigma-Aldrich), was dispensed on the FRTL-5 cells at a concentration of 2 mM one hour before starting the co-treatment with TCDD 1 × 10^−9^ M, for 24 hours.

### Isolation of nuclear/cytoplasmic protein fractions and immunoblotting

FRTL-5 cells were scraped with ice-cold PBS, collected by centrifugation and lysed in hypotonic buffer (20 mm HEPES pH 7.8, 10 mM KCl, 1 mM MgCl_2_, 1 mm PMSF, 0.1% Triton X-100, 20% Glycerol, protease and phosphatase inhibitor cocktail, Roche). Upon incubation on ice for 10 min, cytosolic fractions were prepared by spinning the samples for 1 min at 4 °C at 6000 *g* and subsequently clarified by centrifugation (13,000 *g* for 5 min at 4 °C). The nuclear pellets were firstly washed to remove any residual cytosolic contaminations and, then, lysed in nuclear lysis buffer (20 mm HEPES pH 7.8, 0.2 mm EDTA, 400 mm NaCl, 0.1%, 1 mm PMSF, 0.1% Triton X-100, 20% Glycerol, protease and phosphatase inhibitor cocktail). They were incubated for 20 min at 4 °C with gentle shaking and, finally, centrifuged for 5 min at 4 °C at 15,000 *g*.

Protein concentration was measured by Bradford assay (Biorad Protein Assay) but loaded on gels up to cell equivalent. Nuclear and cytosolic proteins from 1 × 10^6^ cells were separated by SDS–PAGE and transferred onto nitrocellulose membrane. The following primary antibodies were used: rabbit anti-p65 (SantaCruz, sc-372, 1:1000 for p65 determination in nuclear extracts and 1:3000 for cytosolic proteins); mouse anti-β-tubulin (Sigma-Aldrich, 1:5000); rabbit anti-Topoisomerase I (Abcam-Ab109374, 1:1000). Horseradish peroxidase-conjugated secondary antibodies (Amersham Biosciences) have been used for the detection usually diluted 1.5000. Only for the detection of cytosolic p65 it was used diluted 1:10000.

### Animals and treatments

Animal experiments were performed in accordance with the European Council Directive 2010/63/EU following the rules of the D.Lgs 26/14 and procedures were approved by the Ethical committee of the Biogem Institute, Genetics Research “Gaetano Salvatore” (IRGS) and by the Italian Minister of Health (ID number 61-16).

The mice were kept under standard facility conditions and received water and standard diet (#1320, Altromin) or medicated food containing TCDD dosed at 0, 001 μg/kg/day and 0, 1 μg/kg/day (Altromin) “ad libitum”. Food and water consumption was monitored. To expose the animal since the conception, C57BL/6 dams were exposed, 7 days before the mating, to TCDD dosed at 0, 001 μg/kg/day by administration of medicated food. The offspring was exposed by the mothers from gestational day 0 (E0.5) to the weaning (PND21) and, then, by direct feeding till the sacrifice (PND30). In the experiment with genetically modified mice, C57BL/6 females were mated with males of the same strain carrying the double heterozygotic deletion of *Pax8* (*Pax8*^+/−^)^49^ and *Nkx2-1* genes (*Nkx2-1*^+/−^)^50^. Pregnant dams were feed with 0, 1 μg/kg/day TCDD from E15.5. The offspring was exposed through the mother till the weaning and, then, directly exposed by feeding till the sacrifice, at PND60. The consumption of food and water was recorded weekly for all the mice enrolled in the studies. All the animals were sacrificed by carbon dioxide inhalation. Organ of HPT-axis and blood were sampled and properly stored for further analysis.

### fT4 determination and thyroid histology

Venous blood samples were collected in microtubes and centrifuged (5000 rpm for 5 min 4 °C). The serum fraction was kept at −80 °C. Free T4 (fT4) was measured using the fT4 ELISA kit (DiaMetra) following the manufacturer’s instructions. T4-conjugated HRP and TMB substrate system were used for quantification.

For microscopy, thyroids (1 lobe) were embedded in paraffin. 4 µm sections were stained with hematoxylin and eosin (Sigma-Aldrich) according to the manufacturer’s instructions. For immunoperoxidase, target retrieval was performed in TEG buffer pH 9.2, as already described^51^. Briefly, primary antibody anti-p65 (Abcam, ab7970, 1:500) was incubated overnight at 4 °C followed by incubation with the secondary anti-rabbit HRP-conjugated antibody (Dako, P0488, 1:200). Chromogenic reactions were carried out with DAB Peroxidase Substrate Kit (Vector Laboratories). For immunofluorescence analysis, thyroid sections were incubated overnight at 4 °C with anti-p65 primary antibody (Abcam, ab7970, 1:500). Sections were then incubated with secondary Alexa Fluor 488 antibody (Invitrogen); 1 μg/ml DAPI solution was used as nuclear counterstain. Zeiss Axioplan 2 microscope was used for images acquisition (20x and 63x magnification).

### RNA extraction and RT-qPCR

Total RNA, from tissue and cells, was isolated with TRIzol reagent (Invitrogen) according to the manufacturer’s instructions. Reverse transcription, primers design and qPCR were accomplished using QuantiTect Reverse Transcription Kit (Qiagen), NCBI Primer Blast and Fast SYBR Green Master Mix (Applied Biosystems with Applied Biosystem QuantStudio 7 Flex System) respectively, as already reported^22^. Primer sequences are listed in Supplemental Material (see Supplementary Table S1).

Data were normalized by the level of internal control *Gapdh* expression in each sample. The 2^−∆∆Ct^ method was used to calculate relative expression changes.

### Statistical analysis

Statistical analysis were performed using two-tailed Student’s t-test. Probability *p-values* < 0.05 were considered significant. Unless otherwise indicated, at least three independent experiments were considered for *in vitro* data and not less than four animals for each genotype and sex were used for *in vivo* analysis. The results are expressed as the mean ± standard deviation.

## Electronic supplementary material

Supplementary material

## Data Availability

The datasets generated and materials used during the current study are available from the corresponding author C.A. (email: coambros@unisannio.it) or M.D.F. (email: mario.defelice@ unina.it), on reasonable request.
