# Author Correction: Genetic background and window of exposure contribute to thyroid dysfunction promoted by low-dose exposure to 2,3,7,8-tetrachlorodibenzo-*p*-dioxin in mice

**DOI:** 10.1038/s41598-026-60135-3

**Published:** 2026-07-21

**Authors:** Carla Reale, Immacolata Porreca, Filomena Russo, Maria Marotta, Luca Roberto, Nicola Antonino Russo, Emanuele Carchia, Massimo Mallardo, Mario De Felice, Concetta Ambrosino

**Affiliations:** 1 IRGS, Biogem, Via Camporeale, 83031 Ariano Irpino, Avellino Italy; 2 Molecular Medicine and Medical Biotechnologies, University of Naples “Federico II”, 80131 Naples, Italy; 3 IEOS-CNR, Via Pansini 6, 80131 Naples, Italy; 4 Department of Science and Technology, University of Sannio, Via Port’Arsa 11, 82100 Benevento, Italy

Correction to: *Scientific Reports* 10.1038/s41598-018-34427-2, published online 05 November 2018

This Article contains errors.

As a result of errors during figure assembly, in Figure 6a the image for Pax8 ± TCDD (0,1 µg/kg/day) is a duplication of the image for wt CTRL.

The correct Figure 6 and its accompanying legend are shown below.

**Fig. 6:**
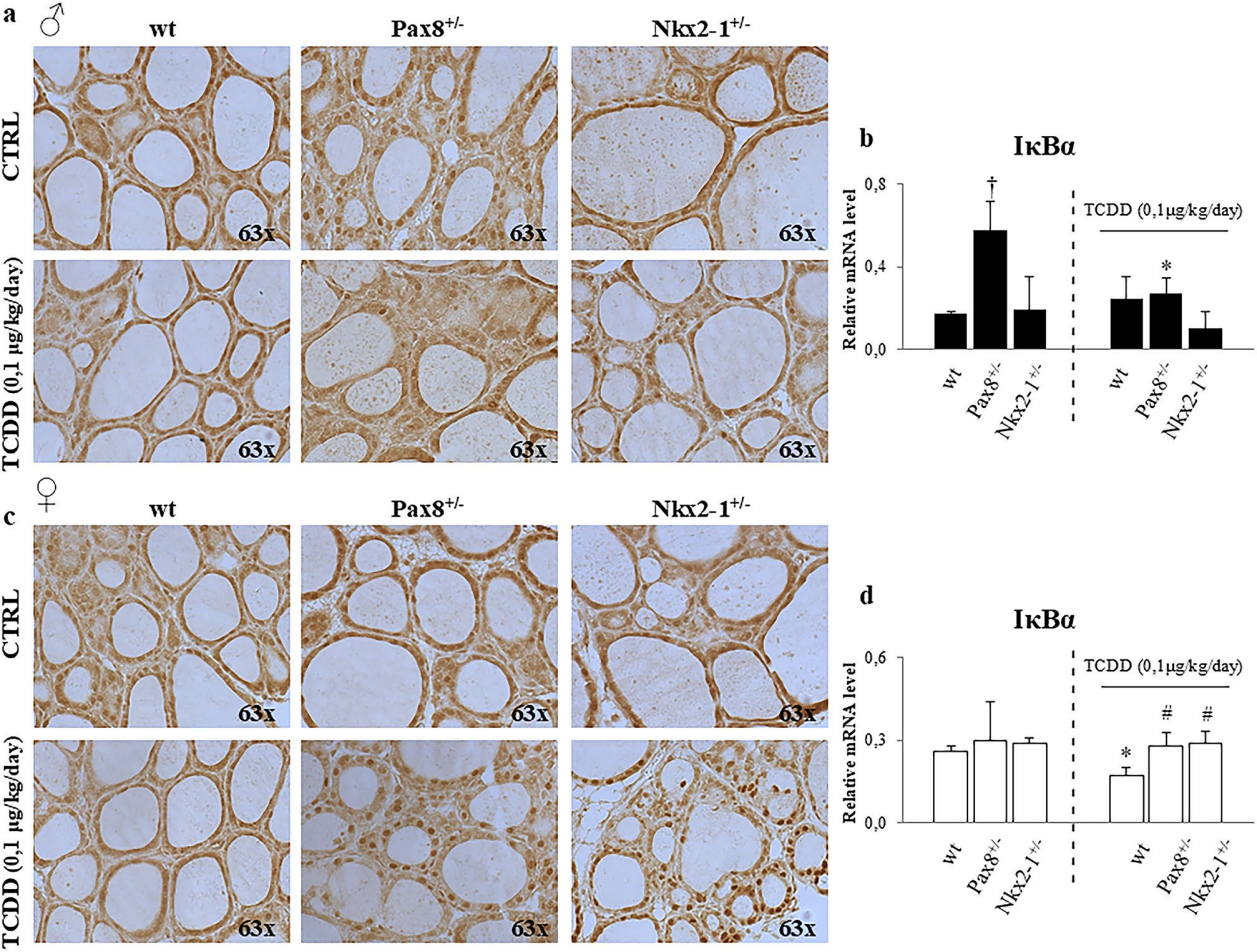
Activation of NF-κB pathway in *Pax8*+/− or *Nkx2-1*+/− exposed mice. (**a**,**c**) Immune-peroxidase analysis of p65 protein in thyroids of CTRL *wt*, *Pax8*+ /− and *Nkx2-1*+ /− and of TCDD-*wt*, -*Pax8*+ /− and -*Nkx2-1*+ /− , males (**a**) and females (**c**) (n = 4 mice for each group and sex). Negative control was performed using purified IgG and it is shown in Fig. S3h. (**b**,**d**) RT-qPCR of *IκBα* gene in thyroid of C57BL/6 wild type (*wt*), *Pax8*+ /− and *Nkx2-1*+ /− (at left of the dashed lines) and in TCDD-*wt*, -*Pax8*+ /− and -*Nkx2-1*+ /− (at right of the dashed lines), males (**b**) and females (**d**). Data are reported as means ± SD of *Gapdh* normalized-mRNA levels; n = 4 mice for each group and sex. *p-value < 0.05 for statistical analysis of TCDD-exposed *vs* untreated mice having the same genotype; ^#^p-value < 0.05 TCDD-exposed *vs* TCDD-wt mice; ^†^p-value < 0.05 unexposed mice of different genotype *vs wt* mice.

