# Supplementary material for: Genetic background and window of exposure contribute to thyroid dysfunction promoted by low-dose exposure to 2,3,7,8-tetrachlorodibenzo-p-dioxin in mice
Source: Sci Rep. 2018 Nov 5;8:16324. doi: 10.1038/s41598-018-34427-2 (PMC6218492; doi:10.1038/s41598-018-34427-2)
Supplement: Supplementary file 1 — Supplementary material [file 41598_2018_34427_MOESM1_ESM.docx]

**Supplemental Material**

**Genetic background and window of exposure contribute to thyroid dysfunction promoted by low-dose exposure to 2,3,7,8-tetrachlorodibenzo-*p*-dioxin in mice.**

Reale Carla, Porreca Immacolata, Russo Filomena, Marotta Maria, Roberto Luca, Russo Nicola Antonino Carchia Emanuele, Mallardo Massimo, De Felice Mario, Ambrosino Concetta

**Supplementary Table S1. RT-qPCR primer sequences.**

| **Gene symbol** | **Species** | **Forward sequence** | **Reverse sequence** |
| --- | --- | --- | --- |
| AhR | Mus Musculus | CTACTCCACTTCAGCCACCC | TGGTACCCCGATCCTCTTGT |
| Cyp1A1 | Mus Musculus | TGTCCTCCGTTACCTGCCTA | TTGGCATTCTCGTCCAGCTT |
| Gapdh | Mus Musculus | GAGTCAAGGGATTTGGTCGT | GACAAGCTTCCCGTTCTCAG |
| IκBα | Mus Musculus | GCACTTGGCAATCATCCACG | CAAGTGGAGTGGAGTCTGCTG |
| Nis | Mus Musculus | TCCACAGGAATCATCTGCACC | CCACGGCCTTCATACCACC |
| Nkx2-1 | Mus Musculus | CGCCTTACCAGGACACCAT | GCTCGAGCTCGTACACCTG |
| Pax8 | Mus Musculus | GCCATGGCTGTGTAAGCAAGA | GCTTGGAGCCCCCTATCACT |
| Tg | Mus Musculus | CATGGAATCTAATGCCAAGAACTG | TCCCTGTGAGCTTTTGGAATG |
| Tpo | Mus Musculus | CAAAGGCTGGAACCCTAATTTCT | AACTTGAATGAGGTGCCTTGTCA |
| Tp53 | Mus Musculus | ATTCAGGCCCTCATCCTCCT | TCCGACTGTGACTCCTCCAT |
| Tshb | Mus Musculus | TGGGTGGAGAAGAGTGAGCGCAT | GGACGGCAGCACTCATGCTTTGA |
| Gapdh | Rattus Norvegicus | CATGGCCTTCCGTGTTCCTA | CCTGCTTCACCACCTTCTTGAT |
| IκBα | Rattus Norvegicus | CCTCTCCATCTTGCCTGTGA | CGTTGACATCAGCACCCAAA |
| Nis | Rattus Norvegicus | TTGTGGTAATGCTCGTTGGC | TCACACCGTACATGGAGAGCC |
| Nkx2-1 | Rattus Norvegicus | GGTGCCCTCTGGCCCTATAG | GCTTGTCGATGATGCCCTTTT |
| Pax8 | Rattus Norvegicus | GGCCACCAAATCTCTGAGCC | GGGAATCGATGCTCAGTCG |
| Tg | Rattus Norvegicus | GGGACTCGTGTGGTAGGCAC | CAGGTTACAAATCAGTGGCCC |
| Tpo | Rattus Norvegicus | ATGGAACTCAGCTATGCAGCC | AAGATGCTGAACAGGAACCGG |
| Tp53 | Rattus Norvegicus | AACCGCCGGCCCATCCT | CTCGAAGCGCTCACGCCCAC |

**Supplementary Figures**

**
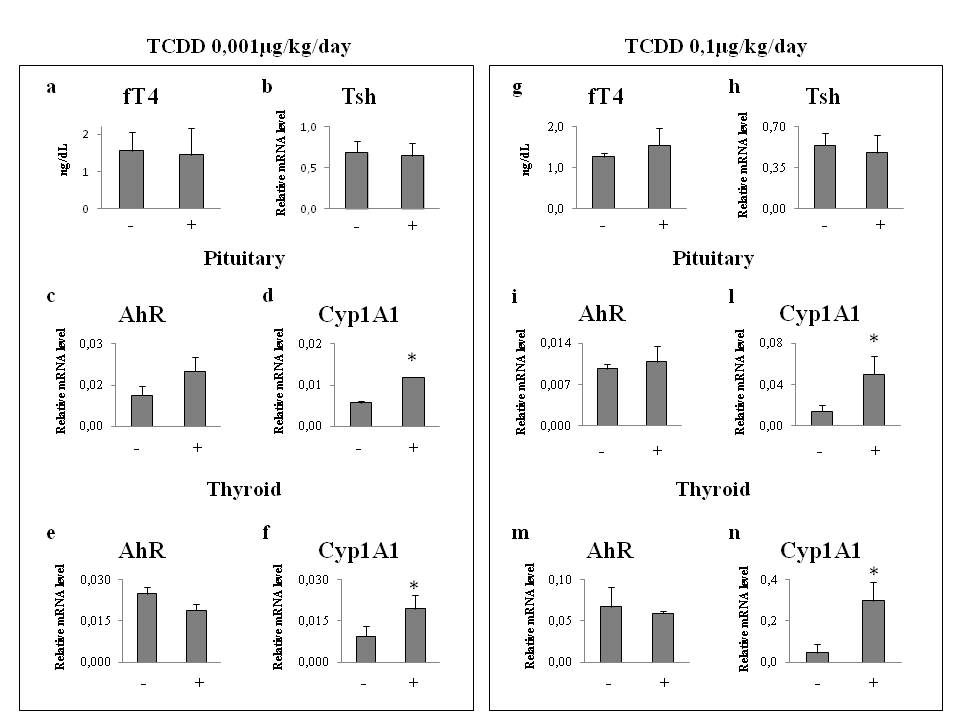
**

**Supplementary Figure S1.** The AhR pathway is activated in thyroid and pituitary of pregnant dams, fed with TCDD-food without impairing of thyroid activity. Pregnant dams were exposed to TCDD 0,001μg/kg/day from the conception (E0.5) at the sacrifice (end of lactation PND21, panels **a**-**f**) or to TCDD 0,1μg/kg/day, from E15.5 at the sacrifice (PND 21, panels g-n) when sampling was conducted. **(a, g)** ELISA assay of fT4 serum level in unexposed (-) or exposed to TCDD 0,001μg/kg/day dams (+, **a**) and 0,1μg/kg/day (+, **g**). (**b**, **h**) RT-qPCR analysis of *Tsh* in pituitary of untreated dams (-) compared to females exposed to TCDD 0,001μg/kg/day (+, **b**) and 0,1μg/kg/day (+, **h**). (**c**-**f**, **i**-**n**) RT-qPCR analysis of *AhR* and *Cyp1A1* transcripts in pituitary and thyroid of TCDD 0,001μg/kg/day (**c**, **d**, **e** and **f**) and TCDD 0,1μg/kg/day (**i**, **l**, **m** and **n**) treated mice. Data are reported as means ± SD of *Gapdh* normalized-mRNA levels; 6 mice for each group were analyzed. *p-value ˂ 0.05.

**
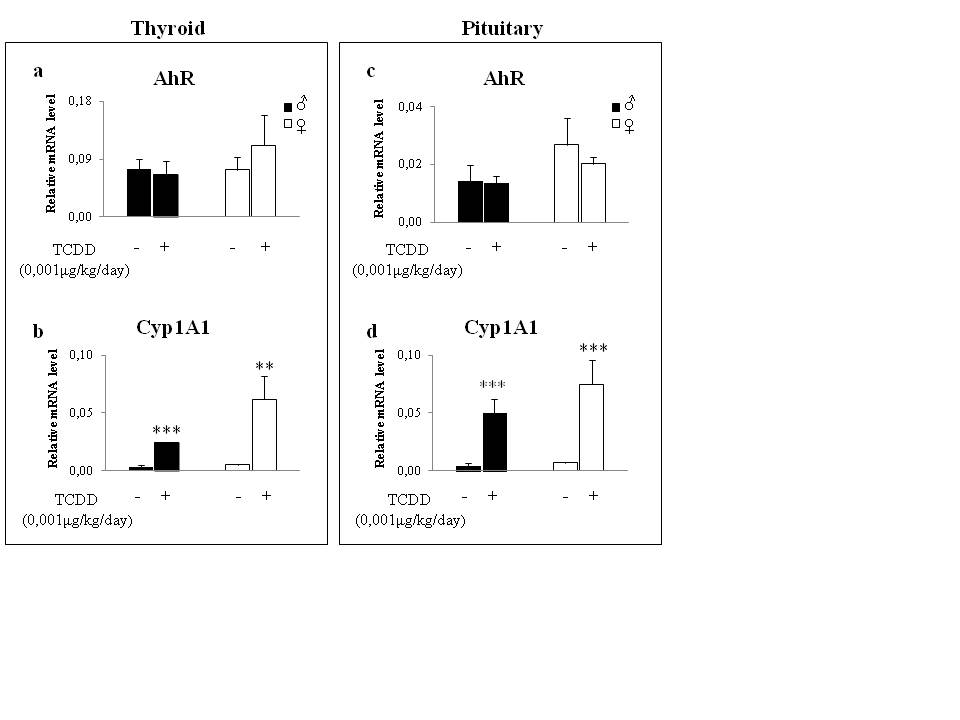
**

**Supplementary Figure S2.** *Cyp1A1* transcript increases in thyroid and pituitary of offspring of both sexes, exposed to TCDD 0,001μg/kg/day from E0.5 to PND 30. Levels of *AhR* (**a**) and *Cyp1A1* (**b**) transcripts were measured by RT-qPCR in thyroids of unexposed mice (-) or treated with TCDD 0,001μg/kg/day (+) of both sexes, male (black bar) and female (white bar). (**c, d**) RT-qPCR analysis of *AhR* (**c**) and *Cyp1A1* (**d**) transcript levels in pituitary of untreated (-) and TCDD 0,001μg/kg/day exposed mice (+). Data are reported as means ± SD of *Gapdh* normalized-mRNA levels of three independent experiments. *In vivo* data are reported as means ± SD of *Gapdh* normalized-mRNA levels; 4 mice for each group and sex were analyzed. *p-value ˂ 0.05; **p-value ˂ 0.01 and ***p-value ˂ 0.001.

**
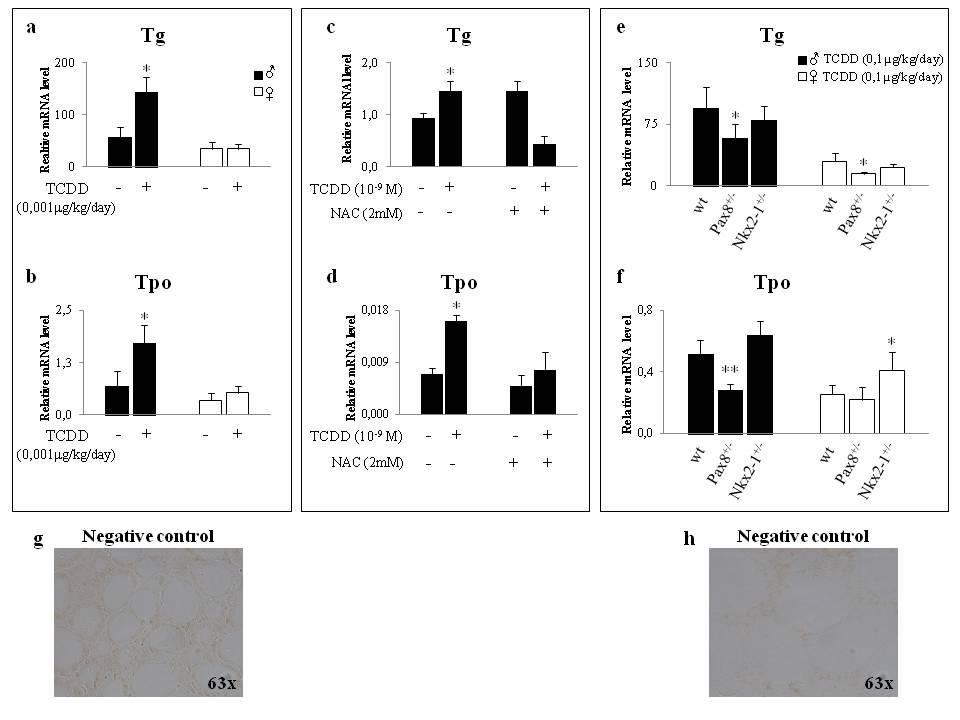
Supplementary Figure S3.** Thyroglobulin **(***Tg)* and thyroperoxidase (*Tpo)* mRNAs expression is regulated by TCDD exposure. *Tg* and *Tpo* transcripts were analysed by RT-qPCR in: C57BL/6 mice of both sexes not exposed (-) or treated with TCDD 0,001μg/kg/day (+) from E0.5 to PND30 (**a** and **b**), male (black bar) and female (white bar); in FRTL-5 cells treated for 24hr with TCDD 10^-9^ M or co-treated with NAC 2mM (**c**, **d**); in *wt*, *Pax8^+/-^* and *Nkx2-1^+/-^* male (black bar) and female (white bar) treated with TCDD 0,1 μg g/kg/day (**e**, **f**). *In vitro* data are reported as means ± SD of *Gapdh* normalized-mRNA levels of three independent experiments. *In vivo* data are reported as means ± SD of *Gapdh* normalized-mRNA levels; 4 mice for each group and sex were analyzed. *p- value ˂ 0.05 and **p-value ˂ 0.01.

(**g**, **h**) Negative controls of immune-peroxidase staining of p65 relative to Fig.2 (**g**) and Fig.3 (**h**).

**
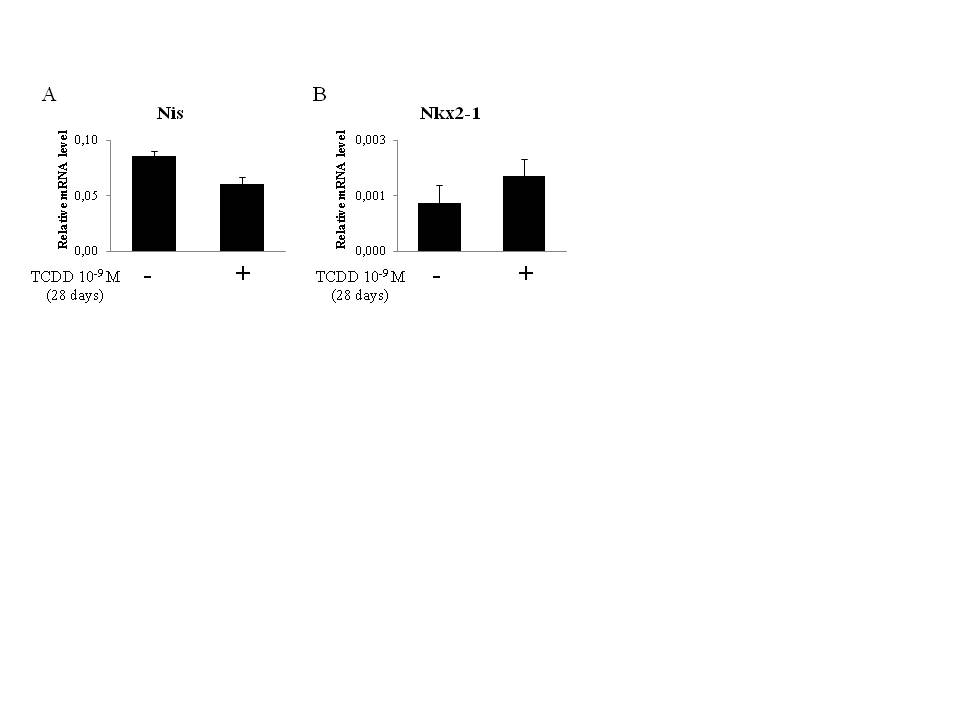
Supplementary Figure S4.** Levels of *Nis* (**a**) and *Nkx2-1* (**b**) transcripts were measured by RT-qPCR in FRTL-5 cells treated with TCDD 10^-9^M for 28 days. Data are reported as means ± SD of *Gapdh* normalized-mRNA levels of three independent experiments.


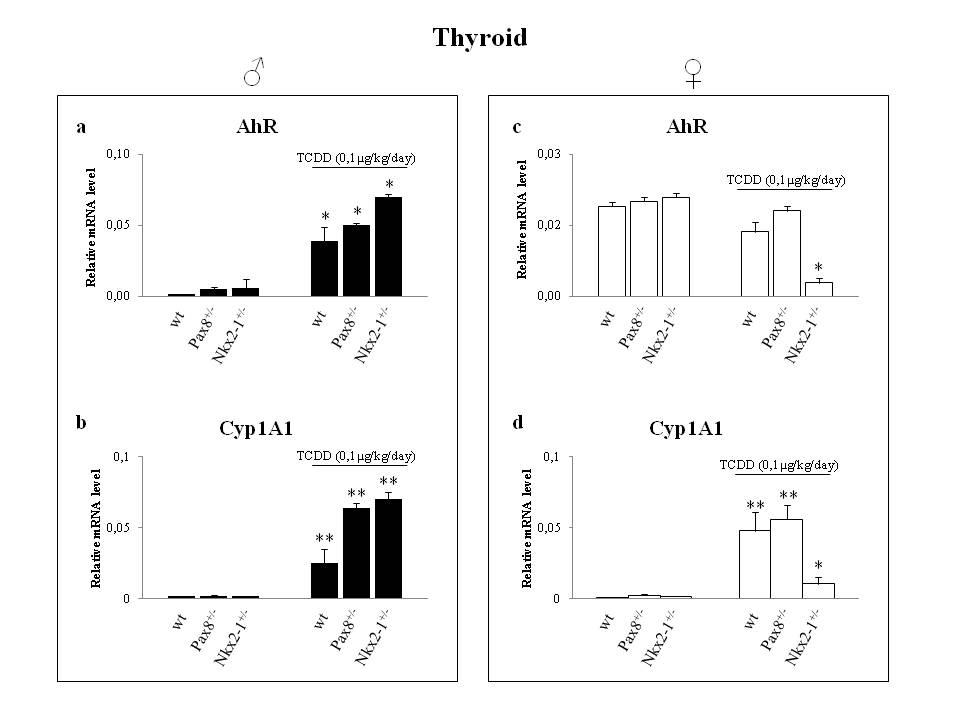


**
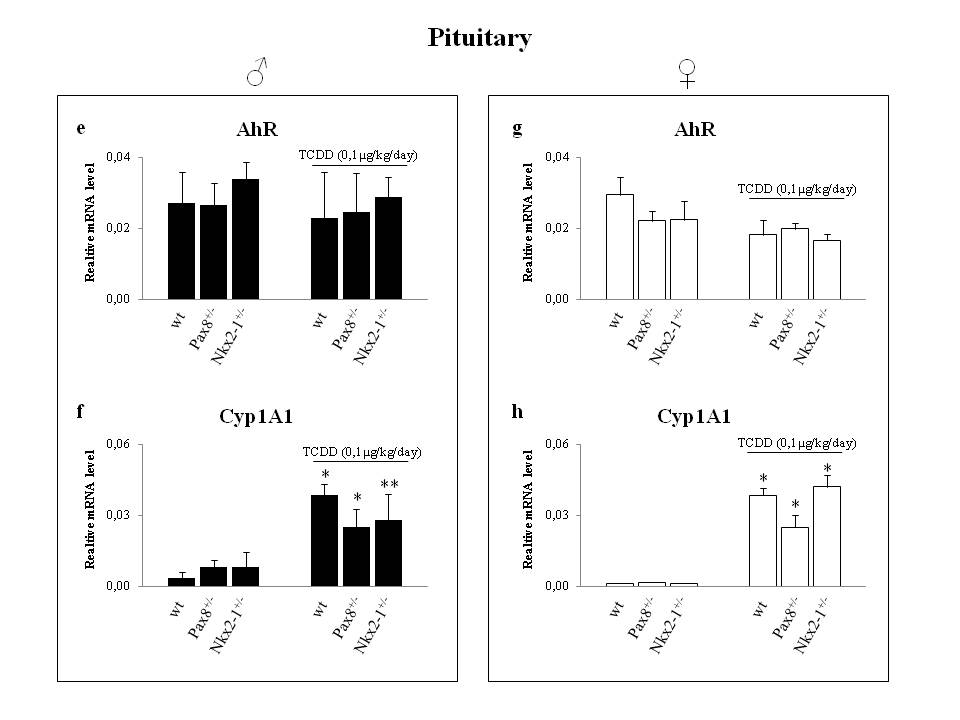
**

**Supplementary Figure S5.** *Cyp1A1* transcript increases in thyroid of the TCDD 0,1μg/kg/day offspring of both sexes exposed from E15.5 to PND60. RT-qPCR analysis of *AhR* and *Cyp1A1* transcripts in thyroid (**a, b**) and pituitary (**e, f**) of wild type (*wt*), *Pax8^+/-^* and *Nkx2-1^+/-^* and TCDD-*wt*, -*Pax8^+/-^* and -*Nkx2-1^+/-^* males. *AhR* and *Cyp1A1* transcripts were also measure in thyroid (**c, d**) and pituitary (**g, h**) of wild type (*wt*), *Pax8^+/-^* and *Nkx2-1^+/-^* and TCDD-*wt*, -*Pax8^+/-^* and -*Nkx2-1^+/-^* of females. Data are reported as means ± SD of *Gapdh* normalized-mRNA levels; 4 mice for each group and sex were analyzed. *p-value ˂ 0.05 and **p-value ˂ 0.01.

**
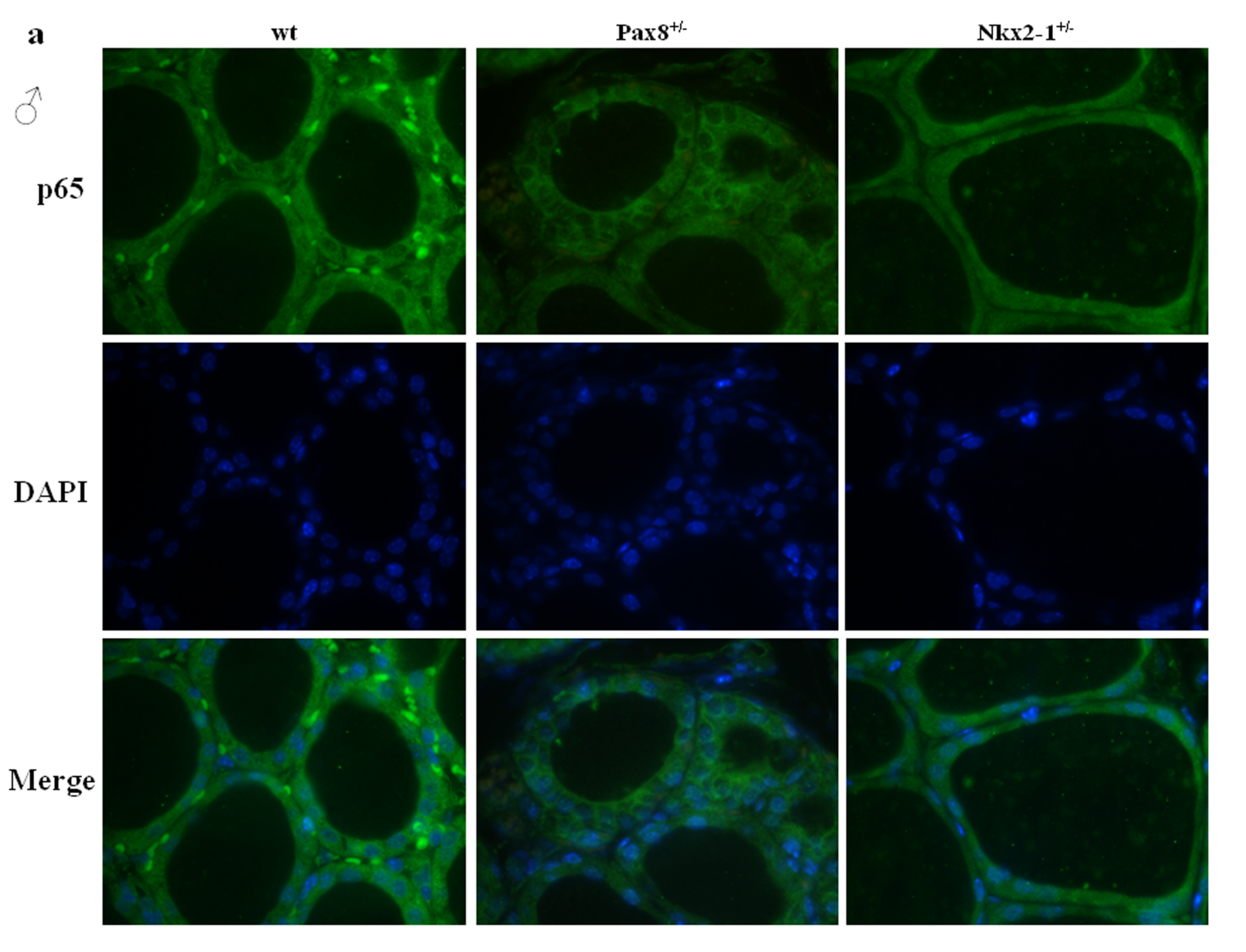
**

**
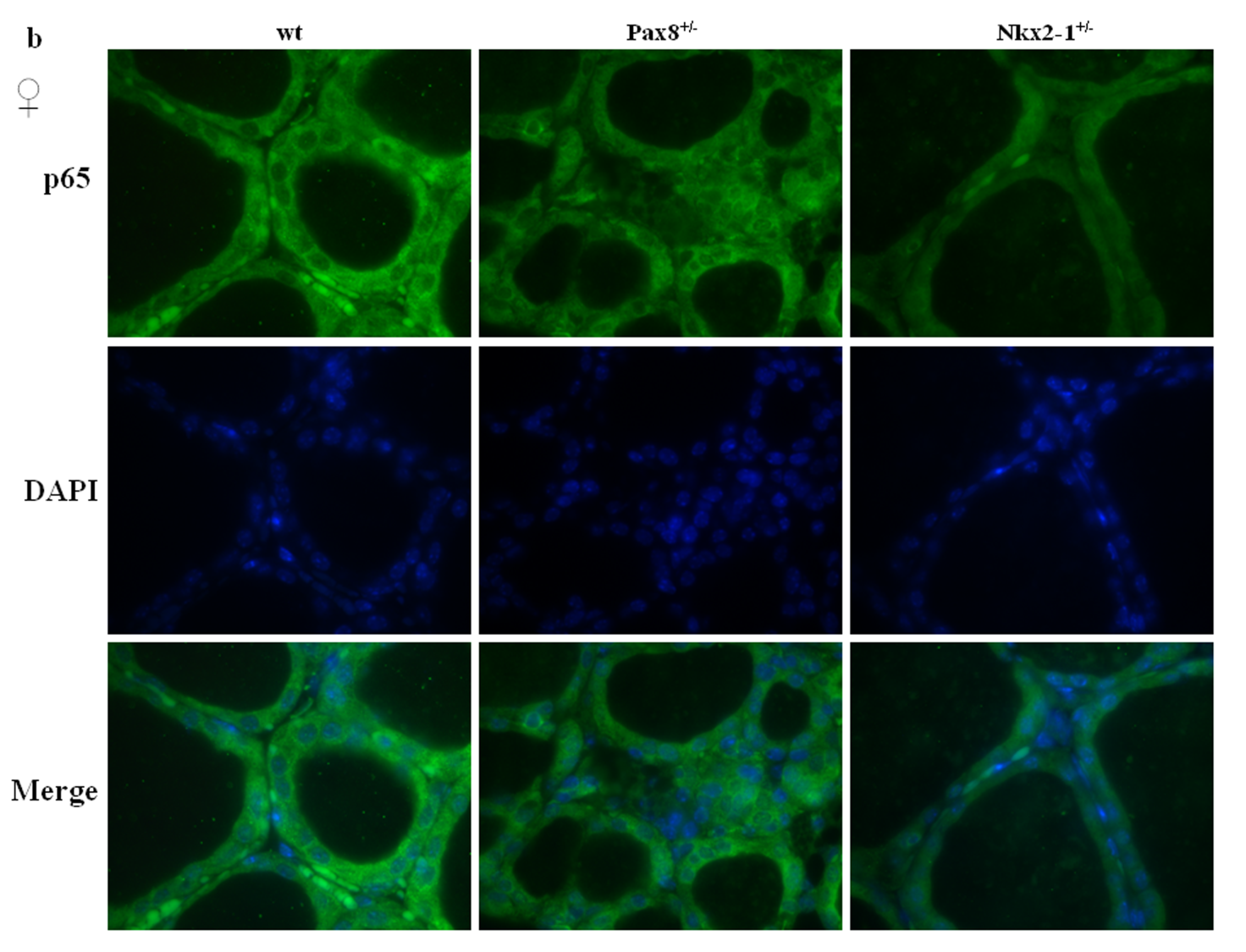
**

**Supplementary Figure S6.** TCDD exposure regulates p65 in genotype- and sex-dependent manner. (**a**, **b**) immunofluorescence of p65 protein on thyroid section from *wt*, *Pax8^+/-^* and *Nkx2-1^+/-^* males (**a**) and females (**b**) mice treated with TCDD 0,1μg/kg/day from E15.5 to PND60. DAPI was used as nuclear counterstain (n=4 mice for each group/sex).


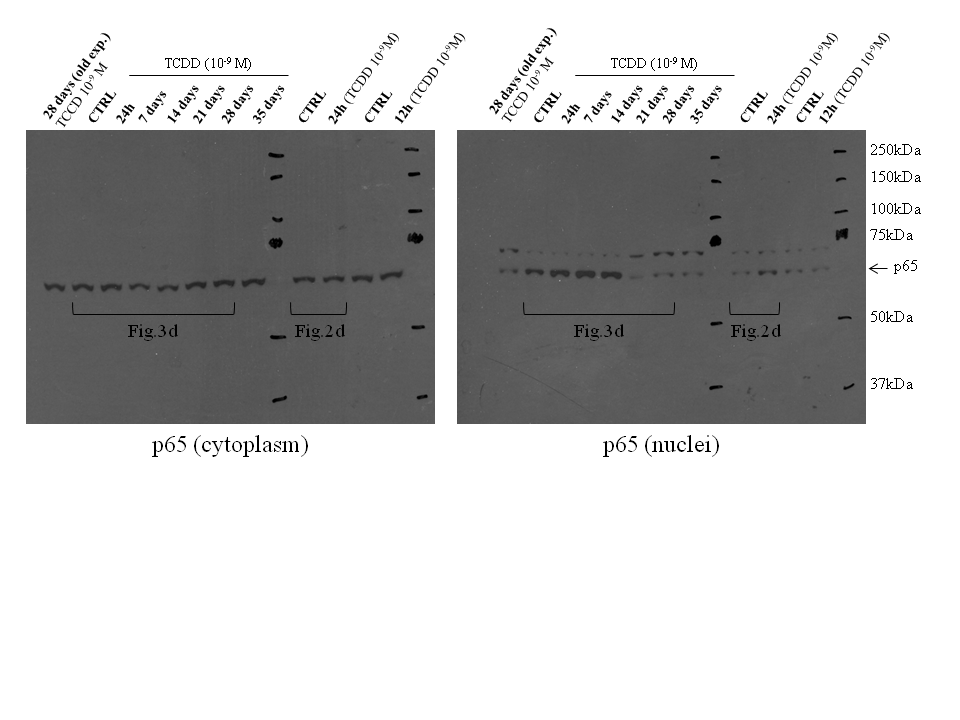

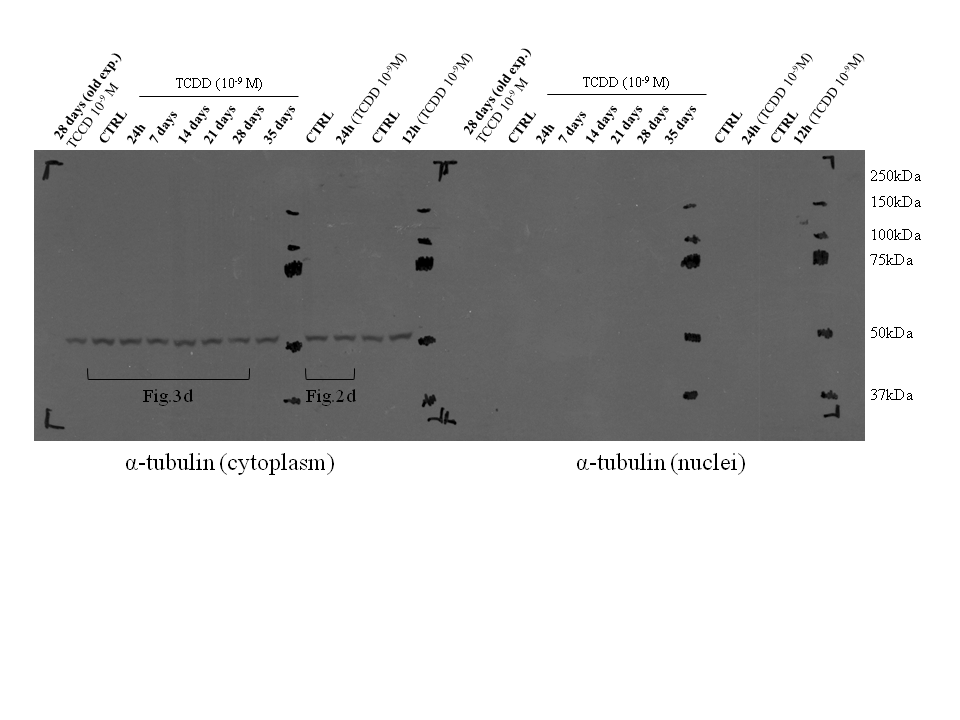

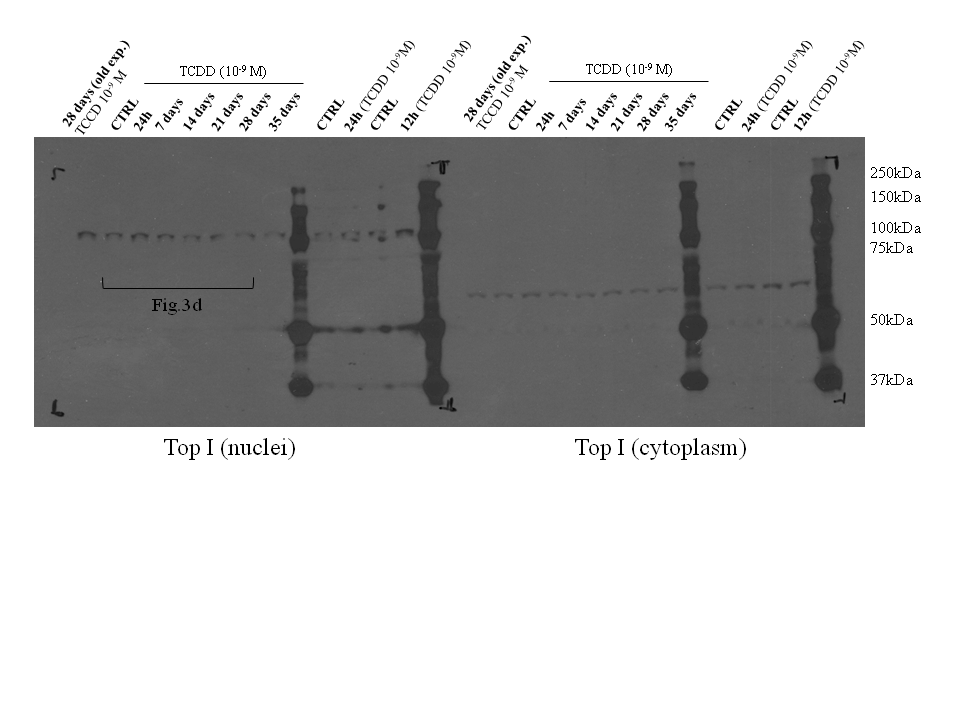


**c**

**b**

**a**


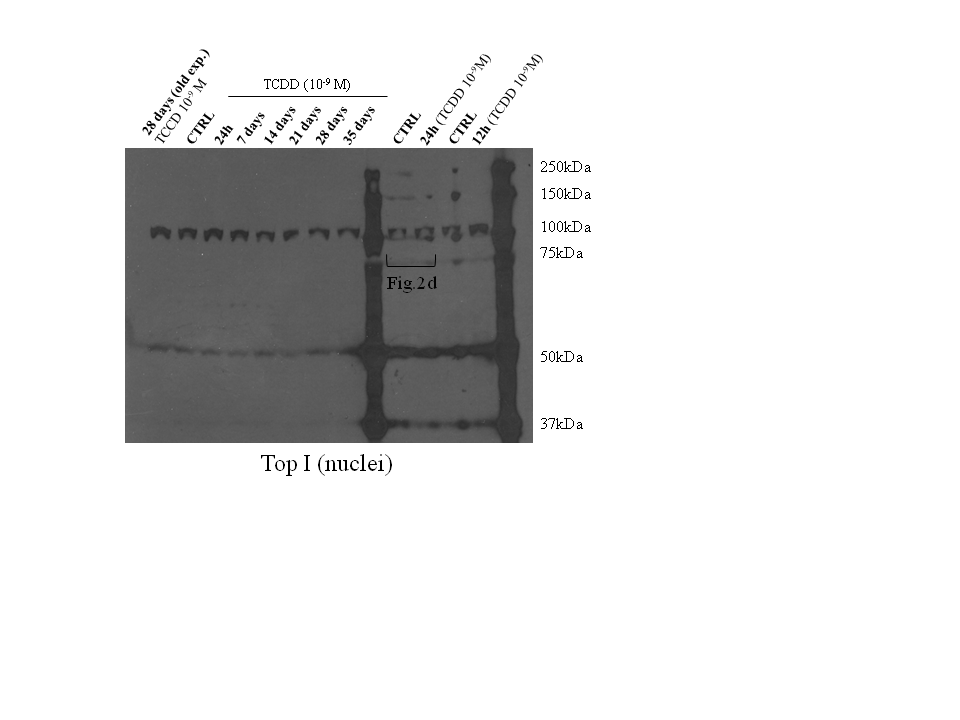
**Supplementary Figure S7.** Original blots. (**a-d**) Full-lenght western blots of p65 (**a**), α-tubulin (**b**), and topoisomerase I (**c**, **d**) proteins. The bands corresponding to the cropped figures 2d and 3d of the manuscript, are indicated. p65 detection was done with two different exposure-time between cytoplasmic (**a**, left) and nuclear fractions (**a**, right), 1 minute and 30 minutes, respectively. (**b**, **c**) α-tubulin and topoisomerase I antibodies hybridization were done in both fractions and detected on the same time, in order to demonstrate no contamination between the two cellular fractions. (**d**) Topoisomerase I of nuclear fraction with different exposure time shown in Fig.2d.

**d**

As it shown, there were loaded other manuscript-unrelated samples: 28 days (old exp.), that is a control of previous experiment made in the same conditions; CTRL, 12h and 35 days of TCDD exposure that we decided to do not include in the analysis.
